# Distribution of Streptococcal Pharyngitis and Acute Rheumatic Fever, Auckland, New Zealand, 2010–2016

**DOI:** 10.3201/eid2606.181462

**Published:** 2020-06

**Authors:** Jane Oliver, Arlo Upton, Susan J. Jack, Nevil Pierse, Deborah A. Williamson, Michael G. Baker

**Affiliations:** Murdoch Children’s Research Institute, Melbourne, Victoria, Australia (J. Oliver);; University of Otago Wellington, Wellington, New Zealand (J. Oliver, N. Pierse, M.G. Baker);; University of Melbourne, Melbourne (J. Oliver, D.A. Williamson);; Labtests Ltd, Auckland, New Zealand (A. Upton); University of Otago, Dunedin, New Zealand (S.J. Jack)

**Keywords:** Streptococcal pharyngitis, acute rheumatic fever, group A Streptococcus, child health, Māori health, Pacific Islander health, bacteria, Auckland, New Zealand

## Abstract

Group A *Streptococcus* (GAS) pharyngitis is a key initiator of acute rheumatic fever (ARF). In New Zealand, ARF cases occur more frequently among persons of certain ethnic and socioeconomic groups. We compared GAS pharyngitis estimates (1,257,058 throat swab samples) with ARF incidence (792 hospitalizations) in Auckland during 2010–2016. Among children 5–14 years of age in primary healthcare clinics, GAS pharyngitis was detected in similar proportions across ethnic groups (≈19%). Relative risk for GAS pharyngitis was moderately elevated among children of Pacific Islander and Māori ethnicities compared with those of European/other ethnicities, but risk for ARF was highly elevated for children of Pacific Islander and Māori ethnicity compared with those of European/other ethnicity. That ethnic disparities are much higher among children with ARF than among those with GAS pharyngitis implies that ARF is driven by factors other than rate of GAS pharyngitis alone.

Acute rheumatic fever (ARF) can cause rheumatic heart disease, which in turn may produce permanent heart damage (*1*). ARF is an autoimmune disease triggered in response to group A *Streptococcus* (GAS) infection. GAS pharyngitis is generally considered the major initiator of ARF, but GAS skin infection may also play a role. Substantial knowledge gaps with regard to ARF risk factors and pathogenesis impair disease prevention and control (*2*,*3*). If GAS pharyngitis is the sole initiator of ARF, then we would expect this infection to be most common in groups in which incidence of ARF is highest. ARF rates peak among children 5–14 years of age (*4*).

ARF and rheumatic heart disease exert a major burden on developing countries. Disease rates are also particularly high among persons of Māori and Pacific Islander ethnicity in New Zealand (*4*–*6*). During 2000–2009, among children 5–14 years of age, ARF incidence among Māori children was 40.2 cases/100,000 children, and among Pacific Islander children, it was 81.2/100,000. By contrast, the incidence for non-Māori, non–Pacific Islander children in New Zealand was 2.1/100,000. Associations between ARF and socioeconomic deprivation have inconsistently been observed (*6*–*10*). During 2010–2013, persons living in the most deprived New Zealand neighborhoods were 33 (95% CI 19–58) times more likely to be hospitalized with ARF for the first time compared with persons living in the least deprived neighborhoods (*6*).

In 2011, the New Zealand government announced a major national Rheumatic Fever Prevention Programme (RFPP), aiming to reduce the national incidence of ARF by two thirds (to 1.4 cases/100,000 persons) by mid-2017 (*11*,*12*). The RFPP strongly emphasized primary prevention of ARF through sore throat management; that is, prompt detection and antimicrobial treatment of GAS pharyngitis before development of ARF (*11*,*12*). In areas with high rates of ARF, the sore throat management aspect of the RFPP had 2 components: school-based throat swabbing clinics and rapid-response primary healthcare clinics (PHCs). School-based clinics operated only when schools were in session. Children with a self-reported sore throat could have their throat swabbed free of charge, either at school or at a rapid-response PHC when certain conditions were met. If the swab sample culture produced GAS, a 10-day course of oral amoxicillin was recommended (*13*). The RFPP was reportedly the largest sore throat management program for ARF prevention ever conducted (*14*). As of 2014, school clinics included ≈50,000 children (*12*). By December 2013, a total of 83% of school clinics had been implemented and additional rapid-response clinics were being set up in PHCs. Public health messages about the value of seeking throat swabbing for those experiencing sore throat symptoms were promoted to populations considered at high risk for ARF (*15*).

The RFPP resulted in a large collection of high-quality diagnostic throat swab sample data, which provided a unique opportunity to describe the distribution of GAS pharyngitis across an entire population and correlate the data with ARF rates. Most RFPP throat swab samples were collected in Auckland, where ≈50% of ARF patients in New Zealand reside (*15*). Our aim was to describe the distribution of GAS pharyngitis in the Auckland population and compare it with the distribution of ARF.

## Methods

### Setting

In 2013, the population of New Zealand was ≈4.5 million persons. The largest city is Auckland, where around one third of the population resides. In the 2013 census, 10% of persons in Auckland identified their ethnicity as Māori, 12% Pacific Islander, 21% Asian, and 57% European/other (*16*). Auckland comprises 3 district health boards (DHBs): Waitemata, Auckland, and Counties Manukau. Many Auckland schools (n = 75) participated in the RFPP school program. Rapid-response clinics were widely implemented (*15*).

The National Health Index (NHI) is a unique patient identifier widely used New Zealand health data; it can be encrypted to protect patient privacy. Demographic information encoded by the NHI includes New Zealand resident status, prioritized ethnicity, sex, birth date, DHB, and New Zealand Deprivation Index (NZDep) score. The prioritized ethnicity classification system allocates persons to a single ethnic group based on a prioritized order of Māori, Pacific Islander, Asian, and European/other. The European/other group refers to non-Māori, non–Pacific Islander, and non-Asian persons (*17*). The NZDep score is an ecologic measure of socioeconomic deprivation corresponding to a neighborhood (*18*). Deciles 1–2 (quintile 1) represent the least deprived neighborhoods, and deciles 9–10 (quintile 5) represent the most deprived.

Persons eligible to have their throat swabbed and receive antimicrobial treatment through the RFPP were Māori and Pacific Islander children 4–19 years of age, all children in that age group living in quintile 5 neighborhoods, and eligible children’s household contacts 3–35 years of age if they visited a school or rapid-response clinic with a self-reported sore throat. Other persons contributed throat swab samples in PHCs when clinicians decided to collect a sample separately from the RFPP (*15*).

### Throat Swab Sample Data Collection

Since mid-2009, the sole community pathology laboratory service provider for the entire Auckland region has been Labtests (*19*). We obtained data on all throat swab samples cultured at Labtests during 2010–2016: patient encrypted NHI, age, date of swab sample collection, sample source (i.e., school clinic or PHC), and culture result (e.g., GAS). Although swab samples collected in school clinics could be distinguished from those collected in PHCs, we could not distinguish between samples collected in rapid-response clinics and those in regular PHCs.

### ARF Data Collection

We obtained data on ARF diagnoses during 1988–2016 from the Ministry of Health (International Classification of Diseases, 10th Revision [ICD-10], codes I00-I02 and ICD International Classification of Diseases, 9th Revision [ICD-9], codes 390–392). We also obtained rheumatic heart disease diagnoses for the same period (ICD-10 codes I05-I09 and ICD-9 codes 393–398). The encrypted NHI was provided for each entry along with the demographic information it encoded. We identified the first ARF entry for each child and removed all later entries. Because ARF precedes rheumatic heart disease in the causal pathway, when identifying initial ARF hospitalizations, we excluded all persons who had been hospitalized for rheumatic heart disease before their first hospitalization for ARF. We excluded from study all admissions for non–New Zealand citizens. We also excluded hospital transfers; thus, only the first record was included for each ARF admission. In so doing, we attempted to limit the dataset to initial presentations of ARF, in accordance with the method adopted by the Ministry of Health in 2013 (*18*). We created a dataset of initial ARF hospitalizations in Auckland during 2010–2016, the initial ARF dataset, and performed basic demographic analyses.

### Statistical Analyses

We performed descriptive epidemiologic analyses according to key outcome measures, stratified according to selected demographic characteristics and whether GAS pharyngitis was detected. We considered a throat swab sample that produced GAS on culture to indicate a case of GAS pharyngitis. Key outcome measures were incidence of throat swab samples (no. samples collected/total no. children sampled), incidence of GAS pharyngitis, and the proportion of total throat swab samples that indicated GAS pharyngitis.

For all analyses, we used R version 3.3.1 (*20*). Because the RFPP was still being implemented in 2013, our later analyses focused on 2014–2016. After 2013, the annual number of swab samples collected peaked, remaining relatively stable with high population coverage. Most analyses concentrated on children 5–14 years of age (*4*). The focus is largely on samples from PHCs because the school programs intensely targeted high-risk populations on the basis of ARF incidence. When calculating seasonal rates, we multiplied numerator (swab) data by 4 to produce annualized rates.

### Rate Calculations

If a person contributed >1 swab sample, that person would be counted >1 time in the numerator. We calculated intercensus population estimates and projections by interpolation and extrapolation, using denominator data from 2006 and 2013 population censuses (*21*). When calculating mean rates, we used the population estimate for the middle of the period. We calculated relative risks (RRs) and 95% CIs for initial ARF and GAS pharyngitis from the number of cases detected in the population. 

## Results

### Settings and Time Trends in Throat Swab Sample Collection and GAS Pharyngitis

**Figure 1:**
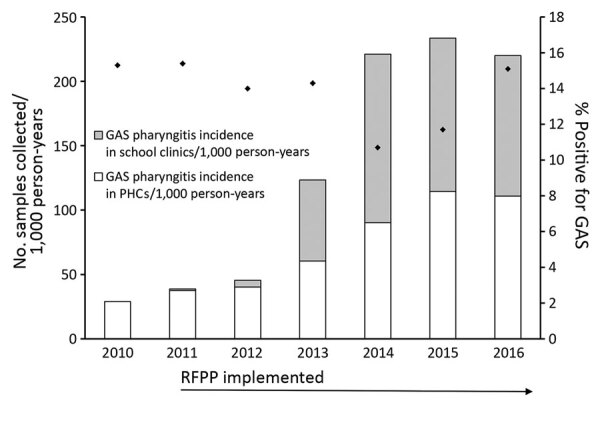
Number of throat swab samples collected and GAS-positive swab samples, by year, Auckland, New Zealand, 2010–2016. Diamonds indicate percentages of swab sample cultures positive for GAS. GAS, group A *Streptococcus*; PHC, private healthcare clinic.

During 2010–2016, a total of 1,257,058 throat swabs were collected in Auckland. The total number of throat swab samples collected each year increased dramatically; 8 times more samples were collected in 2016 than in 2010. During 2011–16, swabbing increased in school clinics but also increased 4-fold in PHCs. The throat swabbing incidence for the Auckland population plateaued in 2014–16; swabbing in school clinics peaked in 2014 (130.8 samples/1,000 person-years) and in PHCs peaked in 2015 (114.5 samples/1,000 person-years; Figure 1; Appendix Table 1).

Of the swab samples, 163,534 were positive for GAS (13.0% total samples); 64,036 were positive for streptococci of group C, group G, or both (5.1% total). The annual proportion of samples positive for GAS decreased from 15.3% in 2010 to 10.7% in 2011 before increasing to 15.1% in 2016 (Figure 1). However, the annual incidence of GAS pharyngitis increased nearly 8-fold from 2010 to 2016. The proportion of positive GAS swab samples was higher among those collected in PHCs (15.0%) than in school clinics (12.6%) (Figure 1; Appendix Table 1).

### Sociodemographic Characteristics of Populations Contributing Throat Swab Samples

We determined the sociodemographic characteristics of populations who contributed throat swab samples in detail for 2014–2016. Sample collection in PHCs was highest among children 5–9 years of age (100,406 total swab samples, 356.6 swab samples/1,000 children), followed by children 10–14 years (72,980 swab samples, 266.5/1,000 children), and then children <5 years of age (68,548 swab samples, 229.1 samples/1,000 children).

**Figure 2:**
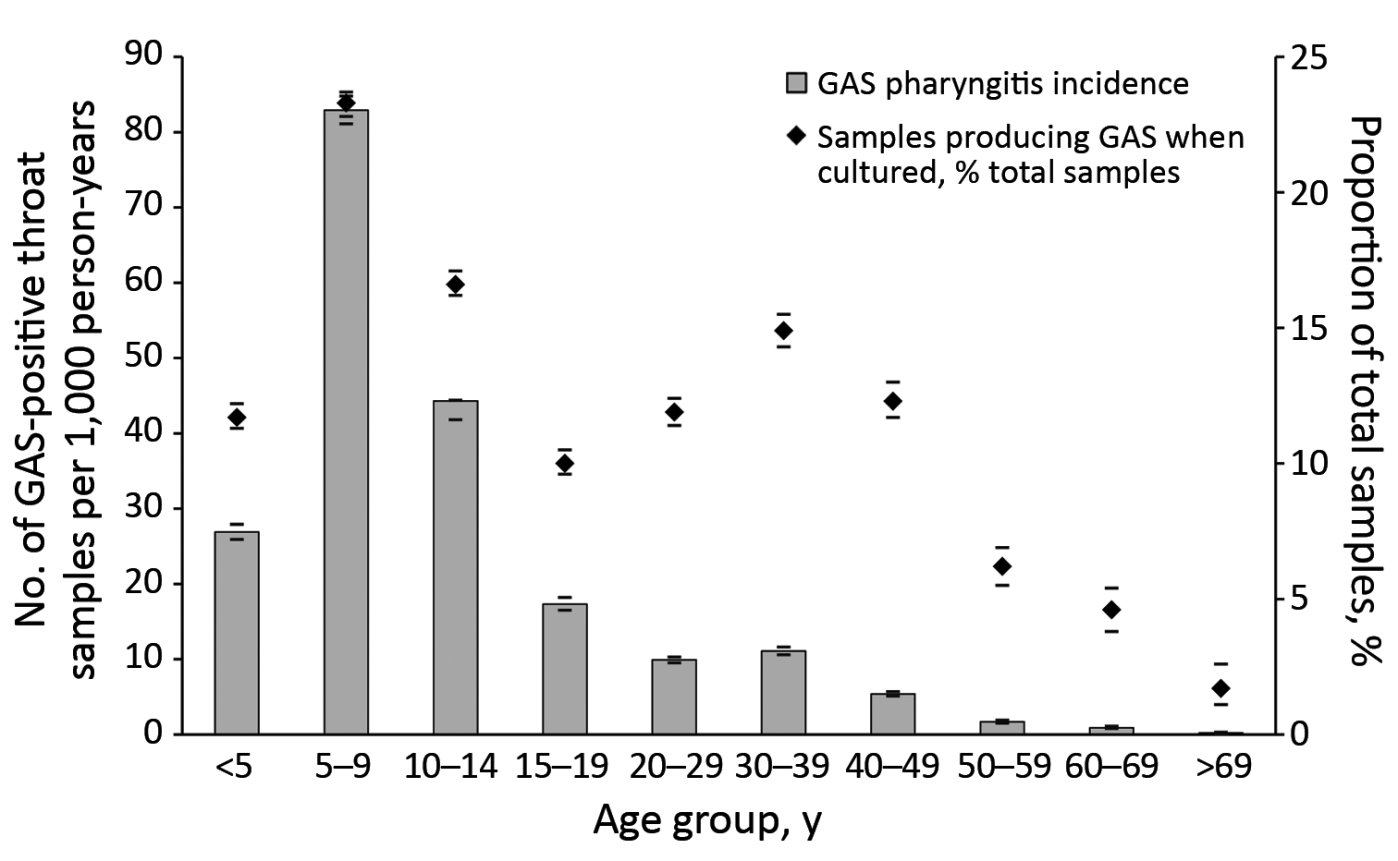
Mean annual distribution of GAS pharyngitis in PHCs, by age group, Auckland, New Zealand, 2014–2016. Diamonds indicate percentages of swab sample cultures positive for GAS; bars above and below indicate 95% CIs. GAS, group A *Streptococcus*; PHC private healthcare clinic.

The incidence of GAS pharyngitis was highest among children 5–9 years of age (82.9 cases/1,000 person-years), followed by children 10–14 years of age (44.3 cases/1,000 person-years). GAS was uncommon in throat swab samples from persons >50 years of age, although ≈15,000 swabs were collected from persons in this age group each year. A much smaller secondary peak in incidence of GAS pharyngitis was seen among adults 30–39 years of age, and 66.2% of those affected were female (Figure 2; Appendix Table 2).

In school clinics, the incidence of swab collection was highest among children 5–9 years of age (1151.3 swabs/1,000 person-years), as was the incidence of GAS pharyngitis (121.8 swabs/1,000 person-years). Of the total school clinic swab samples collected, 96.2% were from children 5–14 years of age, the target age group.

During the study period, 792 persons were hospitalized for initial ARF; 398 (50.3%) were children 5–14 years of age. Because incidence of GAS pharyngitis and ARF were both highest among children 5–14 years of age, we further restricted our analysis to this group. 

### Seasonal Distribution of Swab Sample Collection and GAS Pharyngitis

**Figure 3:**
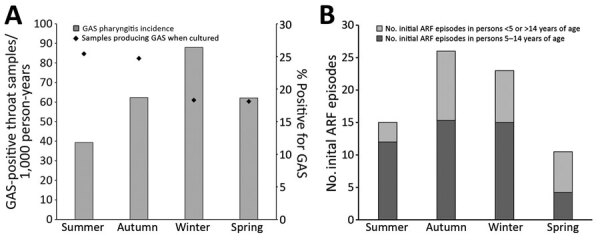
Mean annual distribution of GAS pharyngitis and ARF cases, by season, Auckland, New Zealand, 2014–2016. A) GAS-positive throat swab samples collected from children 5–14 years of age in PHCs. Diamonds indicate percentages of swab sample cultures positive for GAS. B) Mean annual number of first ARF episodes. ARF, acute rheumatic fever; GAS, group A *Streptococcus*; PHC, private healthcare clinic.

The incidence (and RR) of throat swab collection was highest in winter, both in PHCs (480.3/1,000 children 5–14 years of age) and overall; incidence of GAS pharyngitis was also highest in winter (Table; Figure 3, panel A; Appendix Table 3). In winter, the incidence of GAS detected by swab samples collected in PHCs (87.9 samples/1,000 person-years) was more than twice the rate detected by samples collected in the summer. By contrast, the proportion of GAS-positive samples was lower in winter and spring than in summer and autumn. The seasonal pattern of ARF incidence rates was roughly similar to that of GAS pharyngitis; ARF rates were highest in autumn and winter (Figure 3, panel B).

**Table:** Comparison of GAS pharyngitis and ARF rates in the Auckland region, New Zealand, 2014–2016*

Characteristic	GAS pharyngitis in PHCs			GAS pharyngitis in PHCs and schools			Initial ARF hospitalizations	
	Mean annual incidence†	RR (95% CI)		Mean annual incidence†	RR (95% CI)		Mean annual incidence†	RR (95% CI)
Total	15.7	Referent NA		28.1	Referent NA		0.1	Referent NA
Age group, y								
0–4	26.9	2.7 (2.6–2.9)		28.7	2.8 (2.6–2.9)		0.7	0.1 (0.0–0.4)
5–9	82.9	8.2 (7.8–8.6)		204.8	18.1 (17.4–18.9)		17.8	2.5 (1.7–3.8)
10–14	44.3	4.5 (4.2–4.7)		99.7	9.3 (8.9–9.7)		32.5	4.7 (3.3–6.7)
15–19	17.3	1.8 (1.6–1.9)		20.4	2.0 (1.9–2.1)		9.2	1.3 (0.8–2.1)
20–29	9.9	Referent		10.3	Referent		7.1	Referent
30–39	11.1	1.1 (1.1–1.2)		11.5	1.1 (1.0–1.2)		1.7	0.2 (0.1–0.5)
>40	2.5	0.3 (0.2–0.3)		2.6	0.2 (0.2–0.3)		0.0	0.0 (0.0–0.1)
Children age 5–14 y								
Total	62.8	Referent NA		150.6	Referent NA		25.0	Referent NA
M	63.3	Referent		153.4	Referent		25.6	Referent
F	62.5	1.0 (1.0–1.0)		147.6	1.0 (0.9–1.0)		23.7	0.9 (0.7–1.3)
Prioritized ethnicity								
Māori	79.0	1.3 (1.3–1.4)		248.7	3.4 (3.3–3.5)		35.5	86.9 (11.9–635.0)
Pacific Islander	99.6	1.7 (1.6–1.8)		383.4	4.8 (4.7–5.0)		98.3	240.4 (33.5–1,722.6)
Asian	29.2	0.5 (0.5–0.5)		41.0	0.6 (0.6–0.7)		0.0	0.7 (0.0–18.0)
European/Other	58.3	Referent		66.9	Referent		0.4	Referent
2013 New Zealand Deprivation Index quintile								
1	43.5	Referent		45.4	Referent		3.0	Referent
2	39.2	0.9 (0.8–1.0)		45.6	1.0 (0.9–1.1)		2.6	0.9 (0.2–3.8)
3	50.0	1.1 (1.1–1.2)		61.6	1.4 (1.3–1.4)		6.5	2.1 (0.6–7.6)
4	82.4	1.9 (1.7–2.0)		125.7	2.7 (2.5–2.8)		24.7	8.1 (2.8–23.7)
5	103.1	2.3 (2.2–2.5)		427.6	7.7 (7.4–8.1)		76.9	25.2 (9.3–68.5)
Season								
Summer	39.3	Referent		77.0	Referent		25.5	Referent
Autumn	62.3	1.6 (1.5–1.6)		185.3	2.3 (2.3–2.4)		32.6	1.3 (0.8–1.9)
Winter	87.9	2.2 (2.2–2.3)		201.7	2.5 (2.5–2.6)		31.9	1.3 (0.9–1.8)
Spring	62.1	1.6 (1.5–1.6)		138.5	1.8 (1.7–1.8)		8.5	0.3 (0.2–0.6)
District health board								
Waitemata	54.4	1.0 (0.9–1.0)		67.3	0.8 (0.8–0.9)		13.4	0.6 (0.3–0.9)
Auckland	54.9	Referent		80.1	Referent		24.1	Referent
Countries Manukau	72.1	1.3 (1.2–1.4)		270.9	3.0 (2.9–3.1)		36.8	1.5 (1.0–2.3)

*ARF, acute rheumatic fever; GAS; group A *Streptococcus;* PHC, primary healthcare clinic; referent NA, referent not available; RR, relative risk. †Per 1,000 person-years.

### Throat Swab Sample Collection and Incidence of GAS Pharyngitis by Ethnicity

**Figure 4:**
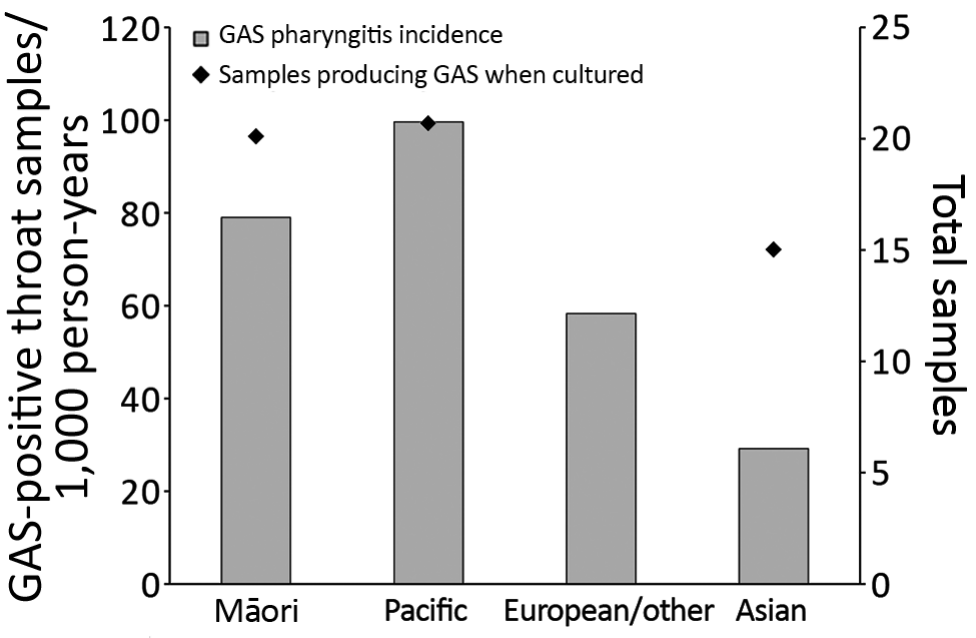
Mean annual distribution of GAS pharyngitis in PHCs among children 5–14 years of age, by ethnic group, Auckland, New Zealand, 2014–2016. Diamonds indicate percentages of swab sample cultures positive for GAS. GAS, group A *Streptococcus*; PHC, private healthcare clinic.

Nearly one quarter of all Pacific Islander children and one fifth of all Māori children contributed >1 swab in PHCs, compared with approximately one sixth of European/other children. The proportion of samples positive for GAS was similar between these groups (20.1%–22.3%), but incidence of GAS pharyngitis was significantly higher among Pacific Islander (99.6 cases/1,000 person-years) and Māori (79.0 cases/1,000 person-years) children compared with those of European/other ethnicity (58.3 cases/1,000 person-years). Incidence of GAS pharyngitis was lowest among Asian children (Figure 4; Appendix Table 3).

### Distribution of Throat Swab Sample Collection and Incidence of GAS Pharyngitis 

**Figure 5:**
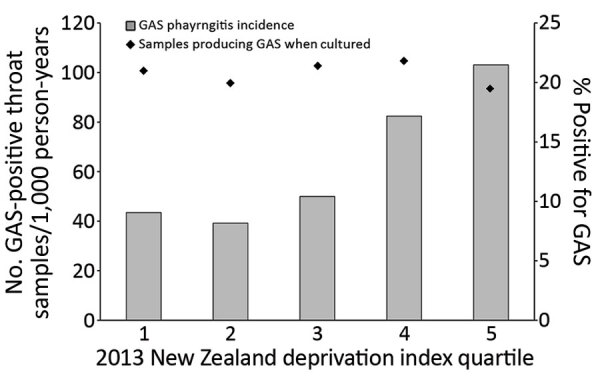
Mean annual distribution of GAS pharyngitis in PHCs among children 5–14 years of age, by NZDep quintile, Auckland, New Zealand, 2014–2016. Diamonds indicate percentages of swab sample cultures positive for GAS. GAS, group A *Streptococcus*; NZDep, New Zealand Deprivation Index; PHC, private healthcare clinic.

Most throat swab samples were collected from children living in the most deprived neighborhoods; >1 throat swab sample was contributed by approximately one quarter of all children from quintile 5, compared with one eighth of all children from quintiles 1 and 2. The proportion of GAS-positive swab samples from children across quintiles was similar (19.5%–21.8%), but incidence of GAS pharyngitis increased with area deprivation. For children in quintile 1, incidence was 43.5 cases/1,000 person-years, but in quintile 5, incidence was 103.1 cases/1,000 person-years (Figure 5; Appendix Table 3).

### Comparison of GAS Pharyngitis and ARF Incidence

We compared mean annual incidence of GAS pharyngitis during 2014–2016, by selected demographic characteristics and swab sample source (i.e., PHC and total), with the mean annual incidence of ARF (Table). The RR for initial ARF was highest among children 10–14 years of age; however, the RR for GAS pharyngitis was highest among children 5–9 years of age. Although ARF was rare among children <5 years of age, GAS pharyngitis was relatively common. The RR for initial ARF was 240.4 (95% CI 33.5–1,722.6) for children of Pacific Islander ethnicity compared with those of European/other ethnicity and was also extremely elevated for Māori children (RR 86.9, 95% CI 11.9–635.0). Of children who contributed throat swab samples in a PHC, the RR for GAS pharyngitis was highest among Pacific Islanders (RR  1.7, 95% CI 1.6–1.8), followed by Māori children, yet this discrepancy was not nearly as extreme as the RR for ARF. Higher RRs for GAS pharyngitis were also estimated for Māori and Pacific Islander children regardless of the sample collection setting. A similar pattern was noted for differences between NZDep quintiles. Seasonality was more pronounced for GAS pharyngitis incidence than for ARF incidence; at PHCs, GAS pharyngitis was least likely in summer and most likely in winter (RR 2.2, 95% CI 2.2–2.3).

## Discussion

The RFPP provided a unique opportunity to assess the distribution of GAS pharyngitis across a well-defined region over a sustained period and compare it with the distribution of ARF. To our knowledge, the RFPP produced the largest ever compilation of throat swab sample data; 1.3 million swab samples were collected in Auckland from 2010 (before RFPP) through 2016. These comprehensive throat swab sample data showed the following: the proportion of GAS-positive swab samples was fairly consistent across the population of children 5–14 years of age but varied between PHC and school clinic settings, suggesting that sample collection thresholds differed by setting; GAS pharyngitis is seasonal and shares some similarities with ARF; unlike ARF, GAS pharyngitis occurs across a wide range of age groups (*4*); and ethnic and socioeconomic differences in GAS pharyngitis are insufficient to explain the marked inequities in ARF incidence.

A striking feature of GAS pharyngitis is the consistent difference in the proportion of GAS-positive samples collected in PHCs and school clinics. The proportion of GAS-positive samples collected from Māori and Pacific Islander children 5–14 years of age in PHCs (20%–21%) was nearly twice that observed for those collected by school programs (11%). At PHCs, the proportion of GAS-positive samples was similar between ethnic groups and NZDep quintiles (except somewhat lower for Asian children). One possible explanation could be that as children approach the level where their caregiver feels they are sufficiently unwell to warrant visiting a PHC, the likelihood of them having GAS pharyngitis is much the same across ethnic groups and deprivation quintiles. Consequently, the proportion of GAS-positive samples by health service may reveal information about the threshold at which persons seek treatment there. Furthermore, the literature describes GAS pharyngitis as a severely painful sore throat (*22*). It is debatable whether many affected children would therefore attend school; many may have visited a rapid response PHC instead. Consequently, GAS-positive samples from school clinics may have largely detected GAS carriage. To support this view, the proportion of GAS-positive samples from school clinics was slightly lower than the estimated prevalence of asymptomatic pharyngeal GAS carriage reported (12%) (*23*). This threshold effect has several implications. First, we should concentrate on findings from PHCs, where the threshold for attendance seems to be higher and a moderate proportion of cases are likely to represent true GAS pharyngitis (not viral pharyngitis with coincidental GAS detection on swab culture) (*24*). Second, GAS pharyngitis incidence rates are likely to provide a better indication of the distribution of this condition compared with the proportion of GAS-positive samples overall. The consistently lower proportion of GAS-positive samples from the school program, at a level equivalent to asymptomatic detection, raises questions about the effectiveness of basing the RFPP in this setting at all. There are potential ways to improve the accuracy of GAS pharyngitis diagnosis, such as through clinical decision rules, although the validity and practicality of such methods are debated (*25*–*28*).

The value of observing the incidence of GAS pharyngitis in PHC settings is illustrated by seasonal distribution patterns. GAS pharyngitis was more common in winter, when incidence was more than twice that in summer. Paradoxically, the proportion of GAS-positive swab samples showed the opposite pattern, being highest in summer. This effect was caused by the large increase in sample collection during winter. Most pharyngitis has a viral cause (*27*); thus, more GAS-negative children reporting a sore throat visited a PHC in winter, reducing the proportion of swab samples that produced GAS in culture. The increase in sample collection during winter was somewhat appropriate given the increased rate of ARF.

Differences in the proportion of GAS swabs across ethnic and socioeconomic groups were insufficient for explaining the marked inequities in ARF incidence rates. Māori and Pacific Islander children, among whom risk of acquiring ARF is highest, were well targeted by the RFPP. Very low rates of ARF in non-Māori, non–Pacific Islander populations in New Zealand have been reported (*29*,*30*), along with ≈200 throat swabs collected/1,000 persons (Appendix Table 3). This observation raises the question of whether intensive swabbing of non-Māori, non–Pacific Islander populations is appropriate. The evidence for reducing swab sample collection from young children is less certain. Although the incidence of GAS pharyngitis was elevated in groups at highest risk for ARF, the age distribution was much broader. If, as hypothesized, ARF is caused by repeated untreated GAS pharyngitis, which eventually triggers autoimmune reactions (priming) (*1*), then concentrating sore throat management strategies on young children is probably justifiable. The extreme disparities in ARF rates between ethnic and socioeconomic groups were not seen for GAS pharyngitis rates. These observations do not support the hypothesis that differences in observed GAS pharyngitis are a key pathway propelling the observed ARF inequities. Consequently, other factors that may drive ARF need to be considered, including the role of GAS skin infections (*31*). Ethnic and socioeconomic differences in exposures to environmental cofactors or host factors also need to be considered as key drivers of ARF inequities (*31*–*37*).

Strengths of our study include complete dataset coverage of a large, well-defined population. Well-characterized numerators and denominators permitted analysis by key demographic attributes. Microbiological analyses were performed by a single provider (Labtests) using standardized protocols. A limitation is that swab sample data reflect healthcare service use rather than representative population sampling, particularly because accurate RFPP coverage data were never collected. In addition, the RFPP deliberately targeted persons in groups at high risk for ARF, for whom GAS pharyngitis risk is potentially higher. These data cannot therefore directly measure the distribution of GAS pharyngitis. A second limitation with this study, and with the RFPP in general, is that it is impossible to know which GAS-positive children have true GAS pharyngitis and which have pharyngitis from other causes and coincidental GAS carriage. No information about clinical manifestations was collected, and serologic confirmation of infection was not sought in the RFPP (and was neither recommended nor practical) (*38*). It is likely that many persons for whom antimicrobial drugs were prescribed had viral infections and may not have benefited from treatment. Last, because the RFPP was not set up to be evaluated, it is impossible to know which swab samples were collected in rapid-response clinics and which as part of routine healthcare. Regardless, the increase in PHC swabbing correlates strongly with, and is most likely attributable to, RFPP implementation (*15*).

A priority for future research is to establish the pathogenic significance of GAS detection in pharyngeal swab sample cultures across different settings. Future analyses could assess the frequency of throat sample collection and GAS pharyngitis for an entire birth cohort of children in Auckland. Population incidence (cohort) studies could be useful for establishing accurate risk measures, particularly of the type conducted in Melbourne, Victoria, Australia, where GAS pharyngitis surveillance data were collected to investigate prevalence, transmission, and serology (*24*). It would be useful to establish an ongoing surveillance and monitoring program for ARF prevention that could assess specific intervention components, such as rapid-response clinics. A case–control study would be well suited to investigate factors contributing to the greatly elevated ARF risk for Māori and Pacific Islander children, beyond exposure to GAS pharyngitis alone (*39*,*40*). Specimens collected in such a study could be used for immune profiling to investigate the hypothesis that cumulative exposure to GAS is indeed a risk factor for ARF (*41*).

In conclusion, we found that the RFPP dramatically increased rates of throat swab sample collection among children at high risk for ARF. Throat swab sample collection is appropriate, given the goal of reducing ARF. However, because GAS pharyngitis is common in human populations, the RFPP resulted in many persons who were not at high risk for ARF undergoing throat swabbing and, for many, antimicrobial drug treatment. The population incidence of GAS pharyngitis shows some correlation with ARF risk. However, disparities in ARF incidence are vastly higher across ethnic and socioeconomic groups than are disparities in GAS pharyngitis, as measured by swab sample cultures from persons with self-reported pharyngitis. This inconsistency implies that factors other than exposure to a single episode of GAS pharyngitis alone must drive ARF development. Identifying and mitigating any modifiable risk factors may hold the key to effective ARF prevention.

Appendix Supplemental results for study of distribution of streptococcal pharyngitis and acute rheumatic fever, Auckland, New Zealand, 2010–2016.
