# Supplementary material for: Distribution of Streptococcal Pharyngitis and Acute Rheumatic Fever, Auckland, New Zealand, 2010–2016
Source: Emerg Infect Dis. 2020 Jun;26(6):1113–21. doi: 10.3201/eid2606.181462 (PMC7258449; doi:10.3201/eid2606.181462)
Supplement: Appendix — Supplemental results for study of distribution of streptococcal pharyngitis and acute rheumatic fever, Auckland, New Zealand, 2010–2016. [file 18-1462-Techapp-s1.pdf]

Article DOI: https://doi.org/10.3201/eid2606.181462

Distribution of Streptococcal Pharyngitis and Acute Rheumatic Fever, Auckland, New Zealand, 2010–2016

Appendix

Appendix Table 1. Throat swabs and GAS detection by year and source, Auckland, total population, 2010–2016

			Swabs collected				
Source	Year	Denominator population	No. swabs per year	Swab rate (per 1,000 person-years)	No. GAS+ve swabs per year	GAS+ve swab rate (per 1,000 person-years)	Prevalence of GAS positive swabs (%)
PHC and School	2010	1314698.6	38145	29.0	5842	4.4	15.3
	2011	1329943.7	51614	38.8	7969	6.0	15.4
	2012	1345188.9	61256	45.5	8580	6.4	14.0
	2013	1360434.0	167776	123.3	24009	17.6	14.3
	2014	1375679.1	304147	221.1	32521	23.6	10.7
	2015	1390924.3	324863	233.6	37853	27.2	11.7
	2016	1406143.7	309257	219.9	46760	33.3	15.1
	2010–2016*	1360434.0	179579.7	132.0	23362.0	17.2	13.0
	2010–2012[1]	1329943.7	50338.3	37.8	7463.7	5.6	14.8
	2014–2016*	1390924.3	312755.7	224.9	39044.7	28.1	12.5
PHC	2010	1314698.6	38145	29.0	5842	4.4	15.3
	2011	1329943.7	49993	37.6	7814	5.9	15.6
	2012	1345188.9	54220	40.3	7920	5.9	14.6
	2013	1360434.0	82266	60.5	12565	9.2	15.3
	2014	1375679.1	124248	90.3	16227	11.8	13.1
	2015	1390924.3	159242	114.5	22179	15.9	13.9
	2016	1406143.7	155903	110.9	27158	19.3	17.4
	2010–2016*	1360434.0	94859.6	69.7	14243.6	10.5	15.0
	2010–2012*	1329943.7	47452.7	35.7	7192.0	5.4	15.2
	2014–2016*	1390924.3	146464.3	105.3	21854.7	15.7	14.9
School	2010	1314698.6	0	0.0	-	-	-
	2011	1329943.7	1621	1.2	155	0.1	9.6
	2012	1345188.9	7036	5.2	660	0.5	9.4
	2013	1360434.0	85510	62.9	11444	8.4	13.4
	2014	1375679.1	179899	130.8	16294	11.8	9.1
	2015	1390924.3	165621	119.1	15674	11.3	9.5
	2016	1406143.7	153354	109.1	19602	13.9	12.8
	2010–2016*	1360434.0	84720.1	62.3	10638.2	7.8	12.6
	2010–2012*	1329943.7	2885.7	2.2	217.6	0.2	7.5
	2014–2016*	1390924.3	166291.3	119.6	17190.0	12.4	10.3

*Mean annual average.

Appendix Table 2. Mean annual PHC throat swabs and GAS detection by age group, Auckland, 2014-2016

	Swabs collected					Individuals swabbed (≥1 swabs/year)				
Age group	Swabs (N)	Swabs/ 1,000 person-years	GAS swabs (N)	GAS swabs/1,000 person-years	Proportion of GAS swabs (%)	Incident swabs (N)	Individuals swabbed/ 1,000 person-years	Individuals with GAS (N)	Individuals with GAS/1,000 person-years	Proportion of individuals with GAS (%)
<5	22849.3	229.1	2683.0	26.9	11.7	14477.3	145.2	2276.7	22.8	15.7
5-9	33468.7	356.6	7782.0	82.9	23.3	18559.0	197.7	6102.0	65.0	32.9
10-14	24326.4	266.5	4040.7	44.3	16.6	14517.0	159.1	3351.7	63.7	23.1
15-19	16996.4	173.0	1704.0	17.3	10.0	11342.0	115.5	1520.7	15.5	13.4
20-29	17836.7	82.9	2121.0	9.9	11.9	13451.0	62.5	1910.3	8.9	14.2
30-39	13486.7	74.7	2002.7	11.1	14.9	10431.3	57.8	1784.0	9.9	17.1
40-49	8814.7	43.7	1085.7	5.4	12.3	7198.7	35.7	986.3	4.9	13.7
50-59	4763.0	27.4	295.0	1.7	6.2	4085.7	23.5	281.3	1.6	6.9
60-69	2572.7	20.4	117.3	0.9	4.6	2246.3	17.8	112.7	0.9	5.0
>69	1349.7	12.2	23.3	0.2	1.7	1204.7	10.9	22.3	0.2	1.9
Total	146464.3	105.3	21854.7	15.7	14.9	97513.0	70.1	18348.0	13.2	18.8

Appendix Table 3. Mean annual PHC throat swabs and GAS detection, Auckland, 5-14 year old children, 2014-2016

	Swabs collected					Children swabbed (≥1 swabs/year)				
Characteristic	Swabs (N)	Swabs/1,000 population	GAS swabs (N)	GAS swabs per 1,000 population	Proportion of GAS swabs (%)	Incident swabs (N)	Children swabbed/1,000 population	Children with GAS (N)	Children with GAS/1,000 population	Proportion of children with GAS (%)
Gender										
Male	27737.3	287.7	6101.3	63.3	22.0	15500.0	160.8	4816.3	50.0	31.1
Female	30056.7	328.2	5720.7	62.5	19.0	16048.7	175.2	4484.0	49.0	27.9
Unknown	1.3	0.0	0.7	-	53.8	1.3	0.0	0.7	0.0	53.8
Prioritised ethnicity										
Māori	12170.7	393.1	2445.0	79.0	20.1	6181.7	199.6	1931.0	61.8	30.9
Pacific	17103.3	480.5	3546.7	99.6	20.7	8605.7	241.8	2783.0	78.2	32.3
Asian	7208.3	194.4	1083.3	29.2	15.0	4400.0	118.7	900.3	24.3	20.5
Other	21313.0	261.5	4747.7	58.3	22.3	12362.7	151.7	3704.7	45.5	30.0
NZDep Quintile										
1 (Low deprivation)	9089.0	207.6	1904.7	43.5	21.0	5425.0	123.9	1500.7	34.3	27.7
2	7509.0	195.6	1505.0	39.2	20.0	4541.3	118.3	1206.3	31.4	26.6
3	7171.0	233.5	1535.0	50.5	21.4	4631.3	150.8	1258.3	41.0	27.2
4	10202.0	377.9	2223.7	82.4	21.8	5780.7	214.1	1762.7	65.3	30.5
5 (High deprivation)	23800.3	527.7	4648.7	103.1	19.5	12065.3	267.5	3663.7	81.2	30.4
Missing	23.7	165.6	5.7	39.8	24.1	14.3	100.1	5.7	39.6	39.9
Season										
Summer	29086.7	154.7	7386.7	39.3	26.9	25554.7	135.9	6884.0	36.6	26.9
Autumn	47414.7	252.2	11714.7	62.3	27.6	38925.3	207.1	10729.3	57.1	27.6
Winter	90288.0	480.3	16517.3	87.9	21.8	68102.7	362.3	14836.0	78.9	21.8
Spring	64392.0	342.6	11672.0	62.1	20.6	51645.3	274.7	10626.7	56.5	20.6
DHB										
WDHB	16481.7	245.7	3650.7	54.4	22.2	9441.0	140.8	2859.7	42.6	30.3
ADHB	12699.3	262.8	2653.0	54.9	20.9	7186.3	148.7	2099.3	43.5	29.2
CMDHV	26313.3	377.2	5030.7	72.1	19.1	13485.3	193.3	3937.0	56.4	29.2
Other	2301.0	-	488.3	-	21.2	1437.3	-	405.0	-	28.2
Mean annual total	57795.3	307.5	11822.7	62.8	20.5	31550.0	167.8	9301.0	49.5	29.5
